# Biochemical sensing in graphene-enhanced microfiber resonators with individual molecule sensitivity and selectivity

**DOI:** 10.1038/s41377-019-0213-3

**Published:** 2019-11-22

**Authors:** Zhongxu Cao, Baicheng Yao, Chenye Qin, Run Yang, Yanhong Guo, Yufeng Zhang, Yu Wu, Lei Bi, Yuanfu Chen, Zhenda Xie, Gangding Peng, Shu-Wei Huang, Chee Wei Wong, Yunjiang Rao

**Affiliations:** 1 Key Laboratory of Optical Fiber Sensing and Communications (Education Ministry of China), University of Electronic Science and Technology of China, Chengdu, 611731 China; 2 State Key Lab of Electronic Thin Film and Integrated Devices, University of Electronic Science and Technology of China, Chengdu, 611731 China; 3 National Laboratory of Solid State Microstructures and School of Electronic Science and Engineering, Nanjing University, Nanjing, 210093 China; 4 School of Electrical Engineering and Telecommunications, University of New South Wales, Sydney, NSW 2052 Australia; 5 Department of Electrical, Computer, and Energy Engineering, University of Colorado Boulder, Boulder, CO 80309 USA; 6 Fang Lu Mesoscopic Optics and Quantum Electronics Laboratory, University of California, Los Angeles, CA 90095 USA; 7 Ubiquitous Sensing Center, Zhejiang Laboratory, Hangzhou, 310000 China

**Keywords:** Optical sensors, Optical properties and devices, Fluorescence resonance energy transfer

## Abstract

Photonic sensors that are able to detect and track biochemical molecules offer powerful tools for information acquisition in applications ranging from environmental analysis to medical diagnosis. The ultimate aim of biochemical sensing is to achieve both quantitative sensitivity and selectivity. As atomically thick films with remarkable optoelectronic tunability, graphene and its derived materials have shown unique potential as a chemically tunable platform for sensing, thus enabling significant performance enhancement, versatile functionalization and flexible device integration. Here, we demonstrate a partially reduced graphene oxide (*prGO*) inner-coated and fiber-calibrated Fabry-Perot dye resonator for biochemical detection. Versatile functionalization in the prGO film enables the intracavity fluorescent resonance energy transfer (FRET) to be chemically selective in the visible band. Moreover, by measuring the intermode interference via noise canceled beat notes and locked-in heterodyne detection with Hz-level precision, we achieved individual molecule sensitivity for dopamine, nicotine and single-strand DNA detection. This work combines atomic-layer nanoscience and high-resolution optoelectronics, providing a way toward high-performance biochemical sensors and systems.

## Introduction

The development of photonic sensors for molecular detection is important for a variety of biological and chemical applications^1,2^. Recently, a series of state-of-the-art sensing schemes have been developed. Taking advantage of the nanoscattering-based mode splitting or shift in microcavities^3–6^, photothermal absorption-excited Fano resonance^7^, evanescent field-enhanced scattering in micro- and nanowires^8–10^ and polymer fiber gratings^11^, interferometric illumination in the dark field^12^, and coherent anti-Stokes Raman spectroscopy^13^, single nanoparticle/molecule detection has been well achieved. Moreover, by breaking the exceptional points formed by non-Hermitian mode degeneracies^14,15^, the sensitivity of microresonator sensors can be further enhanced. Preserving the sensitivity and selectivity is an important consideration for a photonic sensor. To address this, labeling the target is a common approach^16^, while functionalizing the sensor for label-free detection has also been demonstrated in several applications^17–19^.

Graphene and its derived materials have spurred remarkable advances ranging from condensed matter physics, materials science to optoelectronics, mechanics, and biochemistry^20^. Especially in sensing studies, due to their exceptional surface carrier activity, Dirac-Fermion tunability, atomic flexibility, and fast response, graphene material-based devices have provided a platform for biochemical detection^21–27^. Compared with graphene atomic crystals, which have been widely used in devices^28^, graphene oxide (GO) with rich functional groups is more versatile for functionalization^24,29^. However, due to its hydrophilic nature, it is challenging to keep GO films solid on a device.

Here, we introduce a photonic biosensor by depositing partially reduced graphene oxide (prGO) on a capillary-collimated microfluidic dye resonator. The functionalized prGO only interacts with particular target molecules, offering chemical selectivity for the fluorescent resonance energy transfer (FRET)^30^, which provides optical gain for the intermode interferences. The molecular interactions on prGO also induce spectral shifts between the longitudal transmission modes, enabling individual molecule sensing in our high-precision optoelectronic heterodyne interferometry and noise-canceling lock-in amplification system. In experimental implementation, we investigated fundamental biochemical targets, dopamine (a typical neurotransmitter), nicotine (a typical alkaloid), and ssDNA (deoxyribonucleic acid, a long-chain genetic material with rich amino groups), with three types of functionalized resonators. The three species all have a relatively large bonding energy, enabling dye-target exchange in prGO.

**Fig. 1:**
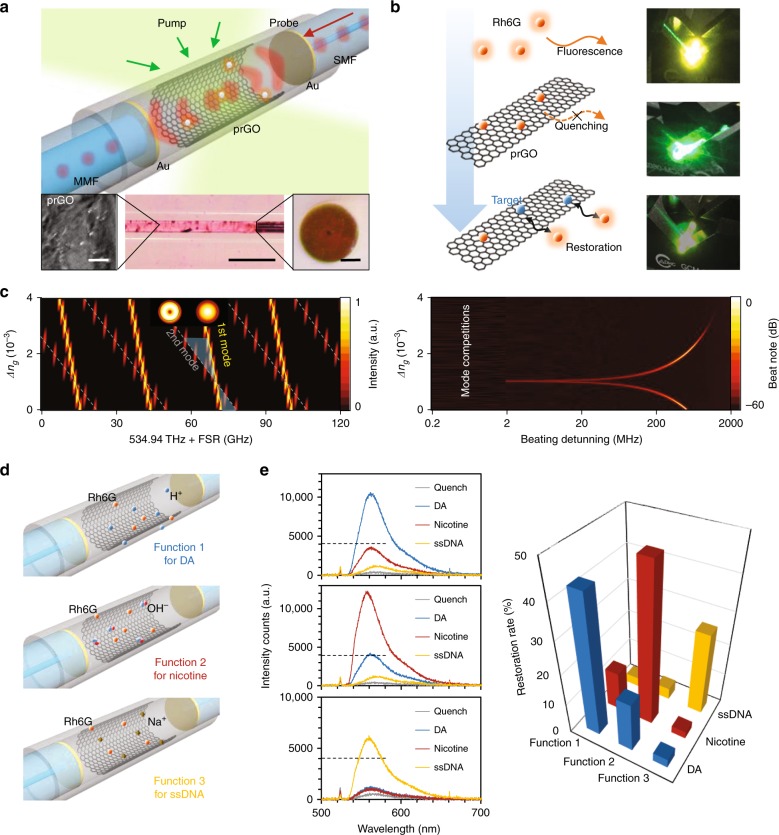
Conceptual design and functionalization of the prGO inner-deposited fiber sensor. **a** Schematic architecture of the device. Two reflectors are collimated in a silica capillary (outer diameter 3 mm, inner diameter 125 μm, and length 10 mm) with the prGO deposited inside. Rhodamine 6G (Rh6G) works as the optical-gain media. Inset from left-to-right shows the scanned electron micrograph of the prGO, the microscopic pictures of the resonator, and the fiber end with Au coverage. Inset scale bars from left-to-right are, respectively, 2, 500, and 50 μm. **b** FRET sensing process. Fluorescence quenches when Rh6Gs (orange dots) attached on the prGO intracavity first, afterwards the targets bind on the prGO, enabling the Rh6G fluorenscent restoration. Here we also show the pictures during this process from top to bottom, with fluorescent color in yellow. **c** In the FP resonator, multiple longitude modes belong to varied *FSRs*. The simulated *FSR*s of the 1st-order transverse mode and the 2nd-order transverse mode are shown, with the resulting frequency shift beat note. Here the thickness of prGO is assumed at 50 nm. **d** Functionalization of the prGO. By linking H^+^ (pH = 2), OH^−^ (pH = 8), and Na^+^ (pH = 7) with the prGO via chemical bonding, FRET in the sensors shows high selectivity detection of dopamine (DA), nicotine, and ssDNA molecules, respectively. **e** Measured results for the sensor-target pairs. Specific pair has fluorescent restoration intensity higher than the others, thereby enabling electronic signal counts over the noise-limited threshold of ≈4000 intensity counts (measurement acquisition time, 1 s).

The all-in-fiber sensor consists of a hollow-cavity Fabry-Perot (FP) resonator, as shown in Fig. 1a. This FP resonator is formed by the end-faces of two collimated standard silica fibers with an external diameter of 125 μm. The input side core diameter is 9 μm, while the output side core diameter is 105 μm for better light collection. Each end facet has been coated with a layer of ≈200 nm gold, which provides over 90% reflectivity at the water/silica interface in the visible band. The highly reflective fiber ends are carefully aligned and fixed by a silica capillary (internal diameter 125 μm), forming a cavity with an end-to-end length ≈4 mm. A uniform prGO multilayer film with a thickness of tens of nanometers is deposited on the inner wall of the capillary via liquid phase reduction and deposition^31^. prGO combines the merits of a crystalline graphene film and GO and is a stable solid in aqueous environments with abundant functional groups, such as hydroxyl and carboxyl groups, based on the chemical reduction degree. More details on the materials and fabrication are in the Methods and Supplementary section 2. The space between the reflectors in the capillary is filled with water containing the dye molecules [we utilized Rhodamine 6G (Rh6G)] and the target biomolecules. A 560 nm LED was used as the probe light for the interference. When pumped with a 514 nm laser, Rh6G provides fluorescent gain from 530 to 660 nm. Before the sensing process, we first attached Rh6G molecules to the prGO film by injecting 100 μM Rh6G solution into the prGO deposited cavity and then dried it. The fluorescence of these Rh6G molecules is fully quenched due to the FRET. Afterwards, when the target biochemical molecules are placed in the cavity, they replace the Rh6G molecules already on prGO due to bonding competition^32^, thus restoring the fluorescence (Fig. 1b and Supplementary Fig. S4). When the restored gain for the probe grows sufficiently strong, the longitudinal modes generated in various resonance families can interfere with each other; then, we can determine the enhanced optical resonance exceeding the noise. We discuss the chemical exchanges and optical-gain assistance in more detail in Supplementary sections 1 and 3. The FP cavity supports multiple optical resonance modes, with mode crossing between the modes. Using the finite element method, Fig. 1c shows the simulated FSRs of the 1st-order transverse mode and the 2nd-order transverse mode in the cavity. When the refractive index of the prGO film *n*_g_ increases by 10^−4^, the spectral distance between the resonances of the 1st and 2nd-order transverse mode changes ≈1 GHz. Hence, such intermode interferences are sensitive to the dynamics of the biochemical molecules due to refractive index modification. In the visible band, the spectral shift of each transverse mode family is below the OSA’s resolution limit (typically, more than 100 GHz), but such a perturbation is clearly seen in the intermode beat note, allowing high-precision electronic measurement discrimination^6^. Different from the mode splitting in high *Q* microresonators due to clockwise and anti-clockwise interference, the intermode interference is in the same direction, staying away from the mode-crossing point, thus enabling a linear response to the effective refractive index. In the experiment, the 514 nm pump laser is in CW operation, providing an average power <200 mW, far below the dye laser threshold^33^. This is advantageous to avoid longitudinal mode competition in the cavity. Figure 1d illustrates specific implementations for detecting dopamine (DA), nicotine, and ssDNA. By carefully adding 3% dilute nitric acid to the DA solution, we maintained pH = 2 in the type 1 cavity with free H^+^. Similarly, by carefully adding 3% aqueous ammonia, we maintained pH = 8 in the type 2 cavity with free OH^−^. The minor H^+^/OH^−^ addition hardly influenced the concentration of DA or nicotine. For ssDNA selectivity, we first immerse the prGO-Rh6G-based cavity in 5% sodium carbonium, dried it and then injected the ssDNA aqueous solution (type 3). More experimental details are shown in Supplementary section 2. Figure 1e plots the chemical selectivity for three investigated species. In our measurements, only the pair ‘function 1 for DA’, ‘function 2 for nicotine’, and ‘function 3 for ssDNA’ demonstrates efficient FRET-based fluorescence restoration. Their fluorescence restoration efficiencies are shown in the histograms. The original fluorescent intensity of 100 μM Rh6G in the resonator is ≈25,000 a.u. The selective response for each functionalization shows threefold or higher discrimination, which enables interferometric measurement with high sensitivity based on the intracavity fluorescent resonances. Moreover, many biomedical applications require a sensor identifying specific molecules in a mixture with more analytes^34^, such as metal ions, glucose, ethanol and cholesterol, such as human blood. In supplementary section 3 and Fig. S7, we verify the selective fluorescence restoration in prGO-Rh6G for these typical analytes for Functions 1, 2, and 3, which can establish the sensor potential to work in a chemical mixture with further functionalization.

**Fig. 2:**
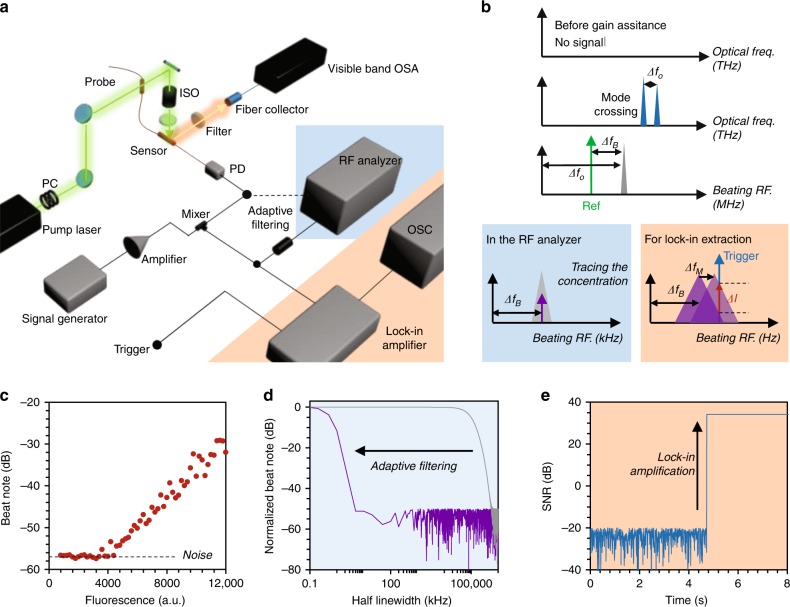
Measurement with enhanced sensitivity. **a** Experimental setup. We mark the optical paths by the green (pump) and orange (Rh6G fluorescence) arrows. The in-fiber laser is collected by a fast photodetector (PD), subsequently analyzed in the measurement electronics. PC: polarization controller; BPS: beam polarization splitter; ISO: isolator; OSA: optical spectrum analyzer; OSC: oscilloscope. **b** Below the threshold, no signal is detected due to prGO quenching, while selective FRET enables measurable optical mode-crossing Δ*f*_O_ (at the MHz level). Beating with the reference sinusoidal signal, the crossing signal loads to a down-converted beat note at Δ*f*_B_ (in the kHz level). In the RF, we track the peak of the beat note and measure its spectral shift (highlighted in the blue box). Through the oscilloscope, we extract and trace the beat note intensity change (Δ*I*) due to the crossing shift (Δ*f*_M_) using lock-in amplification (highlighted in the orange box). **c** The measured RF signals is determined by the fluorescent intensity, wherein the beat note is detectable when the fluorescence is higher than 4000 intensity counts. **d** Frequency response Bode plot based on the half-linewidth comparison. The adaptive filtering in the RF analyzer significantly reduces the beating linewidth from MHz to tens of Hz, enhancing the RF spectral resolution. **e** Signal-to-noise ratio (SNR) enhanced by the lock-in amplifier, with a 44 dB SNR.

To achieve ultrahigh sensitivity, we utilize a two-step optoelectronic heterodyne technique to extract the intermode beating and suppress the optoelectronic noise, as Fig. 2a shows. A stable continuous-wave (CW) 514 nm laser with a maximum power of 200 mW pumps the fiber FP resonator sensor in open space. Related to opto-fluidic dye lasers, which require a pulsed pump with high energy^35^, the CW pumping shows higher stability, both in optics and thermotics, which is critically important for the lock-in measurement afterwards. Moreover, we fix the sensors in a temperature controller for stabilization. On the one hand, we monitored the excited fluorescence of Rh6G by using a visible band OSA. On the other hand, the in-fiber resonant signals are collected by a fast and low-noise silicon photodetector. A broadband scanning electro-signal generator provides a sinusoidal probe with a single Hz linewidth to beat the mode envelope in an electric mixer again for further amplifying and downmixing the beat note for low-pass filtering and satisfying the bandwidth of the lock-in amplifier (sub MHz).

The electronic spectrum analyzer (ESA) with Hz resolution measures the molecular concentration with a large dynamic range, while the oscilloscope (OSC) traces the individual molecule dynamics temporally. Figure 2b describes the scheme step-by-step. For the prGO quenched resonator, there is no optical or electronic signal. When the biochemical molecules restore the fluorescence over 4000 a.u., the detectable threshold is shown in Fig. 2c, the gain assistance overcomes the loss in the cavity; thus, the resonant beatings in the F–P cavity begin to appear over the noise in the ESA. Hence, optical-gain-assisted sensing offers enhanced resolution.

To maximize the spectral resolution, we use the adaptive filtering technique to search the central frequency in the electronic spectral analysis. The Bode plot in Fig. 2d illustrates that the half-linewidth is suppressed from tens of MHz (limited by the gain efficiency and the *Q* factor of the resonator) to tens of Hz (limited by the quantum noise), approaching a four orders of magnitude reduction. As a result, the spectral shift Δ*f*_B_ due to Δ*f*_O_ is quite sharp for the measurement. More information is shown in Supplementary section 1. Filtered by the photodetector, the extracted optical beating (Δ*f*_O_) typically ranges from hundreds of MHz to several GHz, beyond the bandwidth of our lock-in amplifier (125 kHz). Hence, we use a clean sinusoidal probe to beat the Δ*f*_O_ again, generating the second beat note in the kHz band (Δ*f*_B_). The amplification achieves amplitude-frequency demodulation, enabling us to extract the minor signal change due to individual molecule interaction (down to single Hz, corresponding effective RI change 10^−11^ level). As shown in Fig. 2e, locked by an inner triggered reference frequency, the heterodyne interferometer filters the spectral shift-induced intensity alteration at the reference frequency out and enhances the signal-to-noise ratio (SNR) over 50 dB, only limited by the thermal and electronic noise. More detailed discussions are presented in Supplementary sections 1 and 3.

**Fig. 3:**
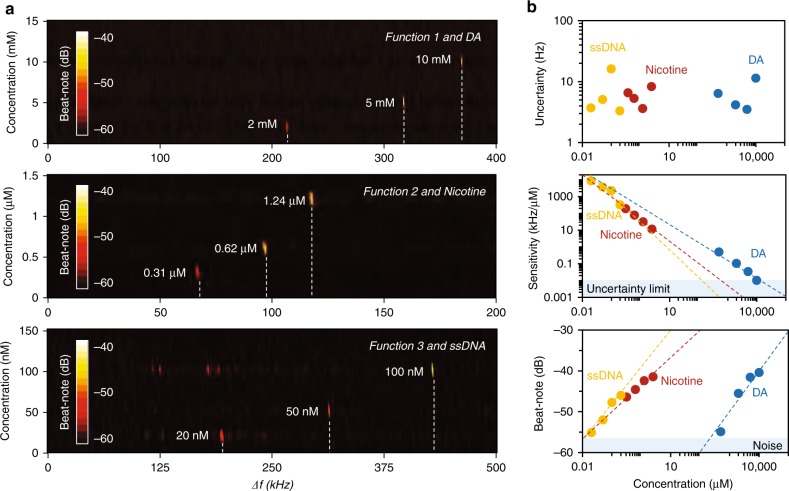
Concentration detection of the molecules based on high-resolution RF beat notes. **a** Measured spectral shifts of the sensor with function 1, function 2, and function 3, for DA, nicotine, and ssDNA detection, respectively. **b** Performance of the sensors. For the three types of target molecules, Allen deviation shows that the RF measurement uncertainty is ≈10 Hz, limited by the electronic noise. For 2 mM DA, 0.31 μM nicotine, and 20 nM ssDNA, our measured sensitivity reaches 0.505, 158, and 8800 kHz/μM, respectively. With increasing concentration, the sensitivity goes down due to the adsorption saturation, thus the dynamic range of the three sensors can approach 10 mM (DA), 0.7 mM (Nicotine), and 0.2 mM (ssDNA), respectively. We note that the higher concentration enables stronger FRET-based restoration.

As a result, in Fig. 3a, we demonstrate the beat note maps of the sensors filled with target molecules at various concentrations. First, we calibrate and normalize the original beat notes without the target molecules for each sample. Then, we measure the spectral shift by adding the target molecules to the specific sensors. For DA at concentrations increasing from 0 to 10 mM, the RF beat of the sensor with *function 1* shifts 365 kHz. For nicotine at concentrations increasing from 0 to 1.24 μM, the RF beat of the laser sensor with *function 2* shifts 110.4 kHz. For ssDNA at concentrations increasing from 0 to 100 nM, the RF beat of the laser sensor with *function 3* shifts 406 kHz (extended data are shown in Supplementary section 3). To investigate the sensing performance, we analyze these results in Fig. 3b. Determined by the laser and filter stability, the spectral uncertainty of all the sensor devices in the electric spectral analyzer is at the ±10 Hz level, which limits the final resolution of the RF measurement. Specifically, for DA, nicotine and ssDNA, the maximum measured sensitivity is 0.51 kHz/μM, 0.2 kHz/nM, and 8.8 kHz/nM, respectively. Moreover, due to saturation of molecular adsorption, the sensitivity will increase when the molecule concentration is lower. The log–log linear fitting approximation shows the sensitivities of the sensors have similar slopes.

When the sensitivity decreases to the uncertainty limit (10 Hz), a further concentration change is undetectable. As a result, the estimated dynamic range of sensors for DA, nicotine and ssDNA sensing is up to 10, 0.7, and 0.2 mM, respectively. By further increasing the prGO surface area and the optical power, the dynamic range can be further promoted. According to the fitted line (for lower concentration, the sensors should have higher sensitivity) and considering the intracavity volume ≈50 nL, these prGO functionalized laser sensors have potential for individual molecule detection. However, determined by the SNR, these sensors cannot detect molecules from zero to one, as we need enough target molecules to meet the detectable threshold overcoming the electronic noise. As the bottom panel in Fig. 3b shows, a lower concentration means a lower beat note intensity (due to lower FRET efficiency), and the detectable limit in the RF is approximately −57 dB. For DA, the detectable amount is 200 μM, while for nicotine and ssDNA, the detectable amount is ~10 nM. Such a difference is mainly determined by the chemical interaction (FRET restoration efficiency) between the Rh6G and prGO, rather than the molecular mass. This limitation induced by the beating intensity shows that it is extremely challenging to achieve ultimate detection by just using RF analysis in these functionalized resonator sensors.

**Fig. 4:**
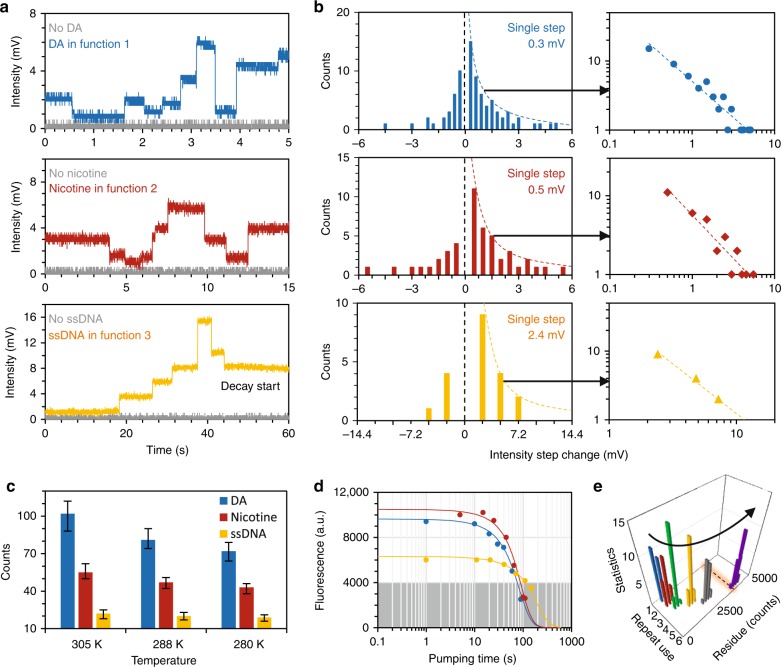
Tracking individual molecular interactions. **a** Discrete changes in these plots correspond to the beat frequency shifts, due to the Rh6G-prGO-target interactions in the FP sensor cavity. The gray curves show the case when there is no target molecule. **b** Statistical histograms show the discrete jumps in the fluorescence counts. After lock-in amplification, for DA, nicotine and ssDNA, the standard deviations are 0.3, 0.5, and 2.4 mV, respectively. The time-bin data obeys the power-law fitting. **c** On/off counts under varied temperature, determined by the Arrhenius equation in principle. The error bars shows the uncertainty in repeated measurements. **d** Bleaching time of the prGO sensors, illustrating the Rh6G bleaching under the pump laser. When the fluorescence is lower than 4000 a.u. intensity counts in OSA, the optical signal is below the dark noise of the PD. Typical bleaching time of the sensing is in the range of ≈100–150 s. **e** Reusability of the prGO-FP sensor cavity. Repeated dye injection degrades the prGO gradually, with a larger quenched residual fluorescence and thus deteriorating the quenching. After five times reuse, the quenched residual fluorescence is larger than the detection threshold (dashed line), disabling the selectivity.

To track the molecular dynamics, we implement the lock-in heterodyne measurement (Fig. 2). Different from the direct spectral analysis in the RF, this does not provide a large dynamic range but extracts and amplifies the miniature intensity alteration of the beat note directly correlated to the reference frequency in a narrow window. In the implementation, the volume of the resonators is fixed ≈50 nL, filled with the target molecules DA (2 mM), nicotine (0.31 μM), and ssDNA (20 nM). Under such a quasi-static environment, the bonding competition among the target molecules, the Rh6G molecules and the prGO would balance dynamically. In Supplementary Fig. S8 and Table S1, we estimate the probabilities of the *target-prGO* interactions analytically. In the resonator, a target molecule attaching onto prGO (exchanging with Rh6G molecules on prGO) results in a discrete signal increment. Conversely, molecular detachment results in a discrete decrement. Figure 4a illustrates the measured results. Target molecules attaching or detaching from prGO bring step-like discrete changes temporally. The integer-multiple relations in the discrete steps suggest the individual molecule dynamics. We note that the discrete steps are induced by the beat note frequency detuning spectrally (Fig. 2b), rather than the Rh6G fluorescence blink photon-by-photon. As a verification, in Supplementary Fig. S8 and Table S2, we demonstrate that the fluorescence blinking on/off of individual Rh6G molecules is nondetectable directly, limited by the photodetector with a typical detection limit ≈−40 dBm. Even in the lock-in measurement, the measured trace demonstrates that the prGO-Rh6G combination is chemically strong; thus, we cannot see any discrete increment due to spontaneous Rh6G release.

We mark these discrete changes over a period of time (1 min) and count the cases in Fig. 4b. For DA in the function 1 sensor, the unit step is 0.3 mV, and the maximum measured step change is 5.1 mV. This corresponds to an increment of 17 units. For nicotine in the function 2 sensor, the single unit step is 0.5 mV, and the maximum measured step change is 5.5 mV. This corresponds to 11 units per increment. For ssDNA in the function 3 sensor, the single unit step is 2.4 mV, and the maximum measured step change is 7.2 mV, which corresponds to 3 units per increment in that time event (such as at the ≈37 s mark of the lowest panel). In the quasi-static state, large steps were rare, whereas unit steps were dominant. These statistical histograms obey the power-law distribution, which is also a sign of individual molecule events^21^. Specifically, we plot the log–log relationship of the increment counts (*C*) based on the discrete intensity change (Δ*I*), fitted by *C* *=* *k/*Δ*I*. For DA, nicotine and ssDNA, *k* equals 5.1, 5.8, and 34.7, respectively. Furthermore, we checked the molecular on/off dynamics by varying the temperature. Determined by the Arrhenius equation, d*M*_a_ = *−*dln*k*/d(1/*T*), the bonding competition tends to be more stronger at higher temperatures. Here, *k* denotes the reaction rate, *M*_a_ is the reaction molecule number, and *T* is the temperature in Kelvin^26^. Figure 4c compares the molecular on/off counts in 1 min when the temperature was controlled at 277, 288, and 310 K. This verification also supports the bonding occurrences.

On the other hand, the performance of the sensors is limited by Rh6G bleaching. As shown in the bottom panel of Fig. 4a, the heterodyne intensity begins to decay when the fluorescence is collected for more than 1 min due to Rh6G bleaching under the strong laser in this measurement. We verified the beaching process by tracing the restored fluorescent intensity of the three types of sensors (Fig. 4d). For the optical sensors filled with Rh6G as the gain media, the fluorescence decreases to <4000 a.u. in ≈100–150 s. This phenomenon is more obvious when the pump energy is higher, which again indicates that CW pumping with proper power is essential. Nevertheless, for each sensing measurement, the FRET process has an intrinsic irreversible residual, as Rh6G tends to permanently attach onto prGO. Although the FRET response of Rh6G here is suitable for prGO-based bonding competition, such bleaching may inevitably limit the performance of the sensors when a longer fluorescence lifetime is needed. On this microfiber resonator platform, other candidates, such as quantum dots^36^, sulfides^37^, and conjugated polymers^38,39^, are also potential fluorescent probes for achieving diverse spectra and high stability. Figure 4b also shows that the probability of molecular attachment on prGO is larger than the probability of molecular detachment. Thus, for these FRET-based sensors, prGO fluorescent quenching weakens with reloading manipulation. Typically, the quenched fluorescence of a device would still remain higher than the detectable threshold after repeated use >5 times, which disables the selectivity of the functionalization and dramatically decreases the sensitivity, as shown in Fig. 4e. This suggests that the prGO/Rh6G-based sensor is a consumable device for in situ detection. Fortunately, the device is composed of all-fiber structures, which is inexpensive, and the cost of each device is ~1$.

Leveraging the unique properties of functionalized prGO nanosheets deposited in a fiber-based multimode dye resonator, we demonstrate a platform for advanced on-line biochemical detection. The functionalized intracavity fluorescent resonance energy transfer enables target selection in the gain-assisted process, and we also achieve individual molecule tracking by measuring the intermode interference in the ultrahigh resolution heterodyne implementations. Such compact, low-cost and network-friendly graphene integrated all-fiber sensors may pave the way for label-free biochemical detection with ultimate performance and provide supplementary devices for medical diagnosis besides in situ methods such as plasmonic resonance^40^ and infrared spectroscopy^41^.

## Materials and methods

### Enhanced sensitivity in the interferometric measurements

Bounded by the measurement resolution in the visible optical frequencies and the high loss in the FP cavity, we inserted optical gain, employed two-step frequency downmixing and transferred the optical intermode interferences to the RF beat notes. We then took advantage of the adaptive peak searching technique in the electronic spectral analysis. In this process, we increased the intensity integration time, suppressing the intensity noise via averaging and fitting the beat notes via the Lorentzian lineshape. Moreover, the dye-based optical gain assisted in enhancing the detectability of the optical mode crossing in FSRs, dependent on the pump power. Keeping the intracavity temperature stable and avoiding immediate bleaching, a higher pump power provides higher sensitivity. More details are described in Supplementary sections 1 and 3.

### Biochemical detection based on the bonding competitions

FRET-based biochemical sensing has chemical selectivity dependent on the bonding competition between Rh6G and DA, nicotine, DNA, and prGO. Rh6G, DA, nicotine, and DNA interact with prGO via its carboxyl and hydroxyl groups. We prepared and diluted the chemical samples from commercial reagents using deionized water. To realize the fluorescent energy resonance transferring (FRET)-based selectivity, we first write the binding of Rh6G and prGO via carboxyl or hydroxyl groups^22^

1 $$({\mathrm{Rh6G}} = {\mathrm{NH}}) + ({\mathrm{HOOC}} - {\mathrm{prGO}}) \to ({\mathrm{Rh6G}} = {\mathrm{N}} - {\mathrm{OC}} - {\mathrm{prGO}}) + {\mathrm{H}}_2{\mathrm{O}}$$

2 $$({\mathrm{Rh6G}} = {\mathrm{NH}}) + ({\mathrm{HO}} - {\mathrm{prGO}}) \to ({\mathrm{Rh6G}} = {\mathrm{N}} - {\mathrm{prGO}}) + {\mathrm{H}}_2{\mathrm{O}}$$

Under critical conditions, external molecules with higher binding energy would bind to graphene instead, replacing the Rh6G molecules; thus, the fluorescence is restored^30^. For pH = 2 (acidic) or pH = 8 (alkaline), Rh6G-prGO reacts selectively with dopamine or nicotine. Moreover, a typical method is to utilize metal ions or nanoparticles as the ribonucleic linker enables DNA-based -NH–NH- bonding^42^. These relationships are shown as follows. More information about the sensing principles and the samples are shown in Supplementary Sections 2.

3 $$({\mathrm{DA}} - {\mathrm{OH}}) + ({\mathrm{Rh6G}} = {\mathrm{N}} - {\mathrm{OC}} - {\mathrm{prGO}}) \to ({\mathrm{DA}} - {\mathrm{OOC}} - {\mathrm{prGO}}) + ({\mathrm{Rh6G}} = {\mathrm{NH}})$$

4 $$({\mathrm{NI}} = {\mathrm{N}} - {\mathrm{CH}}_3) + ({\mathrm{Rh6G}} = {\mathrm{N}} - {\mathrm{OC}} - {\mathrm{prGO}}) + {\mathrm{OH}}^ - \to ({\mathrm{NIN}}^ - {\mathrm{CH}}_3 - {\mathrm{prGO}}) + ({\mathrm{Rh6G}} = {\mathrm{NH}})$$

5 $$({\mathrm{prGO}} - {\mathrm{COOH}}) + {\mathrm{Na}}^ + + ({\mathrm{Rh6G}} = {\mathrm{NH}}) \to ({\mathrm{Rh6G}} = {\mathrm{N}} - {\mathrm{OC}} - {\mathrm{prGO}})^ - {\mathrm{Na}}^{+}\, + \, {\mathrm{H}}^ +$$

6 $$\begin{array}{l}({\mathrm{Rh6G}} = {\mathrm{N}} - {\mathrm{OC}} - {\mathrm{prGO}})^ - {\mathrm{Na}}^ + + ({\mathrm{NH}}_2 - {\mathrm{DNA}})\\ \to ({\mathrm{Rh6G}} - {\mathrm{OC}})^ - {\mathrm{Na}}^ + + ({\mathrm{DNA}} - {\mathrm{NHNH}} - {\mathrm{Rh6G}})\end{array}$$

### Device fabrication and characterization

Standard single-mode silica fibers are used to form the FP cavity, enabling fluorescent interference collection. To achieve higher precision, each fiber end was coated by a layer of gold via vacuum sputtering. Further thermal annealing in an oxyhydrogen flame ensured mirror uniformity. The graphene GO was prepared from natural flake graphite via the modified Hummers’ method. The silica capillary (outer diameter 3 mm, inner diameter 125 μm, and length 10 mm) was held in the GO solution with tweezers vertically until the solution was absorbed into the cavity due to the siphon effect. After drying, the GO in capillary was introduced into the VC solution and heated in an 80 °C water bath. By controlling the concentration of GO and the reduction process, the prGO was uniformly distributed on the inner wall without scarceness or blocking. We note that the prGO film was not a single atomic layer. We also characterized the prGO deposited in the cavity, including Raman spectrum measurements and X-ray photoelectron spectra (XPS) measurements. This verified that the prGO is partially reduced while preserving the key functional groups.

### Experimental set-ups

The visible ion laser (MellesGriot U 4000) offers a stable 514 nm CW pump with stable output and maximum power of 200 mW. A temperature controller (Thorlabs, 10 mK) ensures stable cavity resonances. The visible band OSA (Oceanwave) with a maximum spectral resolution of 2 nm is utilized to check the fluorescence generation, quenching and restoration, assisting the design and functionalization of the dye laser resonator. The silicon-based high-speed PD (Thorlabs, 12 GHz bandwidth, 0.2 A/W) is used to detect the beating envelope. The signal generator (Keysight, 20 GHz) scans the sinusoidal probe frequency to search the beating. The RF analyzer (Rohde & Schwarz) enables RF measurement and triggers from 2 Hz to 43.5 GHz, with a minimum BW of 1 Hz. By using external triggering and internal adaptive filtering, we extract the central frequency of the beat notes down to 10 Hz. With the low-frequency beat note (kHz) sent to the lock-in amplifier (Stanford Research Systems SR830, 125 kHz), we could fix the reference with Hz-level stability. The integral time of the lock-in is 1 ms, and the lock-in amplified signal is viewed with an oscilloscope (Tektronics, 1 GHz, 0.3 mV resolution).

## Supplementary information

supplementary material after proofing

## Data Availability

The data that support the plots within this paper and other findings of this study are available from the corresponding authors upon reasonable request.
