# Supplementary material for: Biochemical sensing in graphene-enhanced microfiber resonators with individual molecule sensitivity and selectivity
Source: Light Sci Appl. 2019 Nov 22;8:107. doi: 10.1038/s41377-019-0213-3 (PMC6874577; doi:10.1038/s41377-019-0213-3)
Supplement: Supplementary file 1 — supplementary material after proofing [file 41377_2019_213_MOESM1_ESM.pdf]

Supplementary Information of

Biochemical sensing in graphene-enhanced microfiber resonators with individual molecule sensitivity and selectivity

Supplementary Materials

Section S1. Theoretical and in-principle analysis
Section S2. Fabrication, characterization and sample implementations
Section S3. Additional methods and extended measured results
Fig. S1. Intracavity multiple transverse modes and their beating interference.
Fig. S2. Processing in electronics.
Fig. S3. Gain assisted resolution enhancement.
Fig. S4. Binding competitions.
Fig. S5. Device fabrication and material characterization.
Fig. S6. Additional fluorescence measurement.
Fig. S7. Comparison of fluorescent restoration of extended analytes.
Fig. S8. Beat notes and lock-in traces in sensing.
Fig. S9. Simulated Molecular distributions in the resonator and measurement of the resonator only filled with Rh6G.
Table S1. Estimation of the molecular activities at room temperature
Table S2. Performance of the measurement
Reference [S1]-[S14]

Supplementary Notes

S1. Theoretical and in-principle analysis

For laser wavelength around 560 nm by using the FRET gain media Rhodamine 6G (Rh6G) solution, the cavity enables multiple transverse modes co-existence. The FSR of a transverse mode is determined by the mode effective index n_{eff}, relying on both the material and the geometry. Figure S1a shows the cross-section of the fiber calibrated FP resonator, composed by a silica capillary. Diameter of the hollow core is 125 μm and when filled with liquid, the refractive index is ≈ 1.345. The inner wall of the resonator is covered by a layer of prGO, with thickness d ≈ 100 nm, and refractive index is 2.2. The silica index is ≈ 1.45. For visible band, such a large cross-sectional area enables multimode transmission. By using finite element method in commercial software *COMSOL Multiphysics*, Figure S1b maps 5 simulated electric field distributions in the cavity, which belong to mode order 1 to 5. On one hand, the higher-order mode has lower field intensity exponentially. On the other hand, each transverse mode has its effective index n_{eff}. From 1st order mode to 5th order mode, the value of the n_{eff} decreases by 5×10^{-5}. Therefore, these transverse modes have diverse longitude mode families, determined by the free spectral range, FSR = $c/2n_{eff}L$. In a 4 mm long FP cavity (L = 4 mm), the *FSR*s of the above 5 order modes are plot in Figure S1c, in a small spectral region (0.3 nm, or ≈ 300 GHz).

The broad fluorescence excite in all the resonance and they would beat each other, obeying the interference relation $f_{beat} = \Delta f_{mode}$. For example, the two-mode beating in temporal domain can be described as:

$$R \times M1 \times M2 = \cos(2\pi f_1 t + \varphi_1)\cos(2\pi f_2 t + \varphi_2) = \frac{R}{2}(A+B) \tag{1}$$

$$\begin{aligned} A &= \cos(2\pi[f_1 + f_2]t + \varphi_1 + \varphi_2) \\ B &= \cos(2\pi[f_1 - f_2]t + \varphi_1 - \varphi_2) \end{aligned} \tag{2}$$

Here t is the time, φ_1 and φ_2 are the initial phases, and R denotes the amplification rate. We call the term A ‘sum frequency carrier’, and the term B ‘differential frequency envelope’. Varying with the frequency, the inter-mode beating due to resonance mismatching are complicated, not only determined by the n_{eff}, but also influenced by the L and the measurement band, as well as the mode competition and nonlinearity [S1].

Typically, for wavelength 500 nm to 700 nm, the mode interference based beating frequency f_1-f_2 ranges in hundreds of MHz to several GHz. Figure S1c simulates the typical longitude mode distributions of the 1st and the 2nd mode in two micrometer space, with different phase velocity. Here we note that limited by the Q factor, resonances have their linewidths, in which signals are band-limited. Referring the Fourier transform, the spectral response of $M1 \times M2$ can be written as the convolution integration $FT(M1)*FT(M2)$, the beat note has the linewidth equaling to the sum of the linewidth of the two optical modes for zero-chirping. Linewidth (Δv) of a resonance is determined by the finesse of the resonator, Here ρ is the fraction that the circulating power left after one round-trip, $f_o = f_1$ or f_2 is the optical frequency.

$$F = \frac{\pi}{2\arcsin\left[\dfrac{1-\sqrt{\rho}}{2^{4}\sqrt{\rho}}\right]} \approx \frac{\pi}{1-\sqrt{\rho}} \tag{3}$$

$$Q = \frac{Ff_0}{FSR} \tag{4}$$

$$\Delta \nu = \frac{f_0}{Q} \tag{5}$$

Accordingly, the optoelectronic detection in the prGO based resonator is the tracking of the mode-to mode spectral shift in frequency domain. Referring that multimode mode excitation physics, the strongest beating interference occurs between the 1st order transverse mode and the 2nd order transverse mode. Related to the fundamental mode distributed in Gaussian shape spatially, the higher order modes have more energy interacting with the prGO film, thus they would be more sensitive to the modification of the prGO film. In optics, the molecular

absorptions in the prGO modulate the optical dispersion (or the momentums) of the mode [S2]. Refractive index of the prGO with rich functional groups is a bit different from the index of graphene, which is just determined by its optical conductivity [S3]. When there are external doping or molecular combinations in the prGO, both the electronic response and the optical response of the prGO changes, especially considering that the prGO film prepared in solution is not monolayer. The index modulation is determined by the chemical potential modulation (carrier launching) and the scale of the absorbed molecules (scattering). Referring the cavity model shown in Figure S1a, we simplify the analysis by linearizing the refractive index of the prGO Δn_g, ranging from 0 to 4×10^4. As a result, effective indexes of the first and the second order mode $n_{eff,1}$ and $n_{eff,2}$, changes respectively around the wavelength 560 nm. We show the calculated result in the top panel Figure S1e in approximation. This leads to resonance shift correspondingly. The bottom panel demonstrates the two major resonances for example.

Besides the resolvability in electronics, the resolvability of the resonances in optics is the precondition of the beating. Due to the periodic FSR, the harmonic beat notes appear in spectrum. Figure S1f shows the schematic distributions. The maximum harmonic range is determined by the *FSR*/2. The resolvable limit is that the linewidth of the resonance has to be smaller than the FSR difference of the two modes at this wavelength (ΔFSR). By using the FEM, Figure S1g simulates the finesse for the 1st order mode and the 2nd order mode, with a fixed roundtrip cavity reflectivity ranging from 60% to 90%. Their FSR difference around 560 nm is about 4 GHz. As Figure S1h shows, another way to avoid spectral overlap is enlarging the ΔFSR, for typical 1.4 GHz beating linewidth (30 finesse), the valley-peak ratio decreases gradually with ΔFSR.

In sensing applications relying on the spectral shift detection, we need to find the beat note peak. Generally a narrower linewidth is preferable. To promote the spectral resolution, a series of methods in optics have been used, such as the implementation of ultrahigh *Q* resonator enabling resonance width down to single MHz (*Q* in 10^8 level), or the excitation of lasers enabling single kHz resolution. Besides the optical methods, peak tracking and adaptive signal regeneration in electronics is another powerful tool for signal extraction. The manipulation to extract the central frequency of the optical beat note is written as

$$N = Findpeak\left\langle \int_0^t filter_{LP}(M1\times M2)dt \right\rangle \quad (6)$$

Here *N* is the extracted signal, *Findpeak* is a function in ESA that search the peak in the detection band. temporal integration averager can cancel the electronic noise and enhance the SNR. $Filter_{LP}$ is the low-pass filter, typically limited by the photodetector. Considering a resonator with finesse

30 (linewidth at 560 nm ≈ 700 MHz), and a typical mode-to-mode spectral distance 10 GHz, Figure S2a shows the scheme, then Figure S2b shows the calculated Bode plot response and central frequency energy ratio in ± 1 kHz band. This enables higher spectral resolution, see Fig. Figure S2c. For lock-in amplification with bandwidth 125 kHz, further frequency down beating is essential. As Figure S2a shows, the second-step beating by using a stable electronic signal generator moves signal *E1* (central freq. GHz) to *E2* (central freq. tens to hundreds kHz), thus the intensity gradient in bandwidth of the *E2* can be filtered out and amplified by using the lock-in amplification scheme. The amplification rate and the lock-in SNR is proportional to the integration time. Figure S2d shows the calculated extraction of the spectral shift of a 700 MHz width signal, a larger spectral shift brings higher intensity difference at the reference frequency (e.g. 100 kHz), and the longer lock-in amplification enables higher SNR. Here we assume the signal is in Lorentz shape spectrally. Moreover, during the amplification process, due to the narrow filtering effect, noise with frequency out of the window (especially the white noise in electronics) is well cancelled.

The optical gain helps to enhance the Q factor in a resonator, via decreasing the optical loss. In principle, the Q factor presents the ratio of the energy stored in the oscillation to the energy dissipated per cycle by damping processes. For a resonator below the laser threshold, its Q factor depends on the optical frequency f_0, the fractional power loss α per round trip, and the round-trip time T_R:

$$Q = f_0 T_R \frac{2\pi}{\alpha} \tag{7}$$

This approximation makes sense when $\alpha << 1$. We note that the Q factor varies with different modes, which have different α and T_R. When assisted by the optical gain, the power loss α can be suppressed, as $\alpha = \alpha_0/g$. Here the α_0 is the linear loss in the cold cavity, g is the gain efficience provided by the spontaneous emission. For 560 nm resonances, Figure S3a plots the calculated correlation of the Q factor and the gain efficiency below the lasing threshold. Referring the linewidth-Q relationship, we also show the linewidth suppression assisted by the optical gain in Figure S3b. As Figure S3c simulates, such a gain effect in the cavity compensates the loss for the resonances, reducing the resonant linewidth, thus helps the resolution enhancement in measurements [S4].

Due to the lack of high resolution fast tunable laser (MHz) or high resolution (sub GHz) OSA for the visible band, we provide the evidence of optical gain assisted sensing by using a similar FP cavity with high reflectors (HRs) and erbium ion solution ($Er^{3+}Cl^{-}_3$) gain media, driven by a 980 nm high power pump. The Er^{3+} gain is not quenchable by the prGO. Besides, a high

resolution tunable laser (500 kHz linewidth, 1510 nm to 1590 nm tunable range) scans the interferometric spectra. The structure is shown in Figure S3d. Figure S3e plots the evolution under varied pump power. In cold cavity, due to low coherence, there is no interference. By increasing the pump power, the resonances appear. Moreover, a higher pump enhances both higher extinction ratio and higher cavity *Q*. Here the HRs are multi-layer DBRs for C band.

In optical sensors, chemical selectivity rely on functionalization in materials typically. To realize the fluorescent energy resonance transferring (FRET) based selectivity, we first write the binding of Rh6G and the prGO via carboxyl or hydroxyl [S5]

$$(Rh6G = NH) + (HOOC - prGO) \rightarrow (Rh6G = N - OC - prGO) + H_2O \qquad (8)$$

$$(Rh6G = NH) + (HO - prGO) \rightarrow (Rh6G = N - prGO) + H_2O \qquad (9)$$

This quenches the fluorescence of the Rh6G, or we say the fluorescent resonance energy transfers to the prGO. Under critical conditions, external molecules with higher binding energy would bind to graphene instead, exchanging the Rh6G molecules out, thus the fluorescence restores, which is so-called binding competition [S6]. This process shows chemical selectivity, also modulates the refractive index of the prGO in the cavity. A simple way to change the binding competition condition of the Rh6G-prGO is tuning the environmental pH value. By preparing pH = 2 (acidic) or pH = 8 (alkaline), the Rh6G-prGO would react with dopamine [S7] or nicotine [S8] selectively

$$(DA - OH) + (Rh6G = N - OC - prGO) \rightarrow (DA - OOC - prGO) + (Rh6G = NH) \qquad (10)$$

$$(NI = N - CH_3) + (Rh6G = N - OC - prGO) + OH^- \rightarrow (NIN^- CH_3 - prGO) + (Rh6G = NH) \qquad (11)$$

Here the DA and NI marks the dopamine and the nicotine. Acidic environment also enables avoiding the DA agglomeration. In nicotine-prGO interaction, the nitride keeps trivalence. These two chemical interactions are shown in Figure S4a and S4b. Here the target is marked by grey, and Rh6G with fluorescent restoration is marked by yellow. Compared to DA and nicotine detection, the functionalization to detect ssDNA is a bit more complicated, as shown in Figure S4c. A typical method is to utilize metal ion or nanoparticle as the ribonucleic linker [S9]. Thus there are two steps in the functionalization and detection process, as the following relationship shows. In Eq. (12), the interaction tends to occur in alkaline environment, thus sodium source like Na_2CO_3 is preferable.

$$(prGO - COOH) + Na^+ + (Rh6G = NH) \rightarrow (Rh6G = N - OC - prGO)^- Na^+ + H^+ \qquad (12)$$

$$\begin{aligned} &(Rh6G = N - OC - prGO)^- Na^+ + (NH_2 - DNA) \\ &\rightarrow (Rh6G - OC)^- Na^+ + (DNA - NHNH - Rh6G) \end{aligned} \qquad (13)$$

S2. Fabrication, characterization and sample implementations

Figure S5a to S5g show the fabrication flow of our prGO based laser sensor. Standard single-mode silica fibers (core diameter 6 μm, outer diameter 125 μm) are used to form the FP cavity, enabling fluorescent interference collection. To achieve the higher finesse, silica-air based Fresnel reflection with ratio 4% is sufficient. We enhance the reflectivity in visible band by coating a layer of gold via vacuum sputtering. Further thermal annealing in hydrogen flame is utilized to ensure the mirror uniformity. Thickness of the uniform gold mirror is ≈ 200 nm, enabling a maximum reflectivity of 90%. A silica capillary with inner diameter of just 125 μm (tolerance 1 μm) encapsulates the dye media and the target molecules in aqueous solution. Before fiber calibration, we reduce the GO to be prGO and deposit the prGO film in the cavity. The graphene GO is prepared from natural flake graphite according to modified Hummers' method. Firstly, 5 g amount of graphite and 20 g of $KMnO_4$ are immersed and stirred at 35 ℃ for 2 h in a 200 mL of concentrated sulfuric acid aqueous solution. Then, 400 mL deionized (DI) water is slowly added into the mixed suspension. The temperature of the suspension is slowly risen and kept at 95 ℃. To reduce the residual $KMnO_4$, 5 mL of H_2O_2 is added into the previous mixture when its temperature decreased to 60 ℃. After adding 100 mL of diluted HCl, the few-layer GO is obtained by washing the suspension several times with DI water and freeze drying.

The silica capillary (outer diameter 3mm, length 10 mm) is washed in an ultrasonic bath of deionized water for 30 minutes to ensure the cleanliness and optical scatteringless. Then the capillary is immersed in the GO solution with a tweezers vertically until the solution is absorbed into the cavity due to the siphon effect. Afterwards the capillary full of solution is placed on the heating plate for drying about 2.5 hours slowly. Then, the capillary is introduced into the VC solution, and heated at a temperature of 80 degrees in a water bath for 20 minutes, and then washed in deionized water to clean the excess VC solution. The process last for roughly 2 minutes. Lastly, we place the device on a heating plate for completely drying (1 hour).

By controlling the concentration of GO and the reduction process, the prGO is uniformly distributed on the inner wall without scarceness or blocking. We note that the prGO film is not a single layer sheet. Thus we inserted two fiber end faces coated with highly reflectivity gold film into the capillary cavity, keeping their end faces paralleled in alignment (error in sub angular second level, controlled by the fiber cutter) to form the prGO-based fiber Fabry-perot microcavity. Before the fiber sealing up, the prGO pre baths in the Rh6G (100 μM) to be the quenched state, via H^+, OH^- and Na^+ functionalization. These H^+/OH^- based functionalization are implemented in the DA/nicotine directly. We use the pH test strips (Mannings, HK) to check the

acidity and alkalinity in approximation. For strengthening the DNA link on prGO, we use $Na^{2+}CO_3^{2-}$ with concentration 5% salt-washing the prGO first, pH value in type 3 cavity ≈ 7.

Compared to graphene, the prGO is rich of functional groups, which enable highly sensitive detections, while related to GO, the prGO can keep being film in water, without aqueous dispersion [S10]. Figure S5h to S5k illustrate the characterizations of the prGO deposited in the cavity, which is fabricated by using our liquid phase reduction technique. As the Raman spectra plots, the prGO keeps the Raman shift of the GO, while both the D peak and the G peak of prGO is narrower than the GO, verifying that the prGO has less defects, for better deposition in the capillary. The X-ray photoelectron spectra (XPS) of the GO and the prGO also illustrates, the 20 minute reduction doesn't destroy the GO, but change the ratio of C-O and C=O bindings. For the GO and the prGO, there are three types of bindings majorly, C=C/C-C (284.8 eV), C-O (286.6 eV), and C=O (288.4 eV). Related to GO, the ratio of carbon atom to the oxygen atom increases from 1.75 to 2.51. In practice, by controlling the reducing time, the reduction degree can be controlled. Optical pictures show our GO, prGO and rGO samples (reducing time 0 min, 20 min, 60 min) have distinguished colors. Moreover, we also show the XPS characterization of the full reduction process in Figure S5k. The C:O ratio of rGO is further higher (3.67). It means there are few functional groups in heavily reduced GO.

In our chemical and biochemical materials preparation, all reagents were of analytical reagent grade and obtained by diluting the stock solution. The R6G powder purchased from J&K Chemical Co. Ltd. was dissolved in deionized water with concentration of 1 mM as stock solution and then diluted with DI water to the concentration of 50 μM to 500 μM for measurements. The dopamine hydrochloride powder was purchased from Yingxin Laboratory and dissolved in DI water down to 1mM. The nicotine was purchased from Yingxin Laboratory(Shanghai). In this experiment, nicotine solution was diluted by DI water with the concentration down to 10mg/L, 20mg/L and 40mg/L. The ssDNA(5'-CTC GAC CGA ATT CGT GGC TCT CAT GAC GGA GA-3') solid was purchased from Sangon Biotech. Co. Inc. and dissolved and diluted by DI water with concentration ranging from 40 μM to 200μM. All the reagents were in gradient configurations for sensing experiment.

S3. Additional methods and extended measured results

Figure S6a shows the measured pump absorption spectrum and the gain spectrum of our dye media (Rh6G), we measure the fluorescent spectrum in 560 nm to 580 nm majorly. Figure S6b shows the picture of the fiber calibrated FP resonator in the holder, with temperature control, which helps to suppress the thermal noise. Figure S6c shows the fluorescent quenching of 100

μM Rh6G on the prGO, and a typical restoration by using the nicotine (in pH = 8). Generally, the prGO induced quenching is adequate, with ratio over 20 dB. This enables the selectivity in principles. In Fig. S6d we demonstrate that the prGO based quenching and restoration efficiency varies with the concentration of the Rh6G in the pre-implementations. When the concentration of the Rh6G higher than 100 μM, the prGO cannot fully quench the fluorescence, signals can still be detectable in the RF spectrum, thus weakens the selectivity. On the other side, when the concentration of the Rh6G is too low, target adsorption based fluorescent restoration is not obvious, thus in sensing measurement we cannot see the regenerated signal. Limited by the photodetector noise, the electronic maximum SNR is -50 dBm, many high-order notes are non-detectable.

We provide more information about the performance of the sensors in practice as well. For fluorescence based optical devices, bleaching can be a problem that limits its lifetime. During the pumping process, because the thermal damages or the photon absorptions change the chemical properties of the dye, the bleaching brings irreversible degradation to the gain media (Rh6G). Higher laser power brings faster beaching. That is also a reason why we do not use the high energy pulsed laser pump, nor further increase the resonator Q to million level, as some rare-earth ion doped cavity achieved. Figure S6e shows the measured fluorescent spectra duration of the 100 μM Rh6G, pumped by the CW 514 nm laser with fixed power 80 mW. Such a beaching also reflects in the lock-in amplified trace. Besides, it is known that the fluorescent restoration is <100%, typically <50% in our cavities, that means the Rh6G molecules attached on the prGO film is not completely removed from the prGO film. For repeatedly loading Rh6G, limited by this prGO-Rh6G on-off recoverability, the quenching efficiency decreases. When the prGO is fully filled by the Rh6G, the sensors cannot perform selectively. For repeatedly target loading, the fluorescent restoration rate also goes down. Such a result is due to the passivation of the prGO. The repeatedly measured quenching rates and restoration rates are shown in Figure S6f.

In practical applications, biochemical sensors are always in-need to work in mixtures such as blood serum and urine with other analytes inside. Besides DA, nicotine and ssDNA, we also investigate the fluorescent restoration performance of our three functionalized types, for Na^+ (commonly existing in body fluid), Cd^{2+} (a typical heavy metal), glucose, ethanol and cholesterol (typical biomarkers). All the analytes are prepared in aqueous solution. Figure S7a to 7c show the restoration ratios of Function 1, 2, and 3. Here concentration of Na^+, Cd^{2+}, ethanol, glucose, and cholesterol are 1 μM, 1 μM, 10 mM, 10 mM, and 10 mM, respectively. These numbers are in relatively high level in blood serum. It reveals that function 1 and 2 would be cross-sensitive to other materials like glucose, while function 3 shows better identification to their targets.Brief

explanation of the results: 1) Function 1 with pH = 2 inhibits ion attachment on prGO due to excessive H^+ [S10]. 2) Functional groups in glucose, ethanol and cholesterol are majorly methyl and hydroxyl, which is hard to link on prGO directly. To detect them based on prGO, inducing metal ions or metal oxides is in-need [S11, S12, S13].

After energy integration, find-peak-regeneration and further frequency down mixing by using the signal generator, we show the RF spectra in sensing processes in Figure S8a to S8c. During the sensing, we keep the reference electronic signal fixed, we regard the original points at the fundamental beat note frequencies in the Rh6G filled resonators, without quenching. This corresponds to the results shown in the main text Fig. 3. Here the small random peaks appear on the left of the signal are induced by the thermal noise or related mode competitions. Compared to the RF measurement based on signal regeneration and beating, single molecule tracking asks for higher resolvability and sensitivity, thus the signal integrates longer in the lock-in amplifier. Accuracy and reliability of this measurement is determined by the temporal stability in the sensors [S14]. Helped by the TEC, uncertainty in the cavity and the electronic system is in 10 Hz level, which also limits the filtering width of the lock-in amplification, allowing maximum integration time 100 ms. Moreover, determined by the relatively long integration time, it is difficult for the lock-in measurement to track ultrafast on-off cases of the molecules. Hence we measure the intra-cavity molecular interaction with quasi-static state, by keeping the cavity temperature near room temperature. For example, Figure S8d compares the oscilloscopic traces of the lock-in amplified signal in the free-running system and the thermally stabilized system (288 K), when detecting the molecular dynamics of 2 mM DA. Figure S8e compares the cases when using several different integration time, under the same temperature (288 K). Either too small amplification or too long integration hinders the observation of the individual molecule interaction cases.

By using the finite element method, we simulate the quasi-static distributions of the molecules (2 mM DA, 0.31 μM nicotine, 20 nM ssDNA) in the fluidic resonator, Figure S9a. In the simulation, molecular distribution in space is determined by the Brownian motion (random moving) majorly. For quasi static state, the spatial distribution of molecules is uniform in probability. Compared to the cavity (mm^3), scale of the target molecules are orders smaller (nm^3). We can regard the target molecules as 'points' with gravitation approximately. The DA-prGo bonding is weakest, while the ssDNA-prGO bonding is strongest. In the simulation, we fix the temperature 288 K. Table S1 illustrates the estimated numbers.

Finally, we show the case when there is only Rh6G in the resonator. To enable intermode interference, we launch sufficient Rh6G (1 mM). Figure S9b illustrates the measurement scheme. We measure the optical signal collected by the photodetector by using electric power meter directly, as well as by using the lock-in amplification. Figure S9c plots the measured output optical power of the resonator. Referring the typical PD gain efficiency 0.2 A/W and dark current 150 nA, the optical power estimates 22 μW, with Q limited FWHM ≈ 70 MHz. Fast photonic quantum fluctuation is not detectable just by using a power meter, but the fluorescent bleaching is obviously gradual. In the lock-in measurement, the integration time is 1 ms, correspondingly the effective amplification bandwidth is 1 kHz. Hence we estimate the locked-in optical energy 1874 eV. Hence, referring Figure S9d, the scale in the OSC corresponding to the amplified signal could be 137 eV/mV in maximum. For 560 nm wavelength (≈ 535.7 THz), single photon energy is around 2.74 eV. That means, the sensitivity of the lock-in amplification in the OSC can reach 50 photons per mV ideally. Referring the measurement uncertainty 0.2 mV, the lock-in technique cannot count photon number one-by-one. Table S2 summarizes the discussion above. This comparison verifies that the discrete steps shown in Fig. 4 are caused by the bonding competition rather than the intrinsic blink of Rh6G.

Supplementary References

1. Bahl, G., Zehnpfennig, J., Tomes, M. & Carmon, T. Stimulated optomechanical excitation of surface acoustic waves in a microdevice. *Nat. Commun.* **2,** 403–406 (2011).
2. Shivananju, B. N. *et al.* The roadmap of graphene-based optical biochemical sensors. *Adv. Funct. Mater.* **27,** 1603918 (2017).
3. Vakil, A. & Engheta, N. Transformation optics using graphene. *Science* **332,** 1291–1294 (2011).
4. Chen, W., Özdemir, Ş. K., Zhao, G., Wiersig, J. & Yang, L. Exceptional points enhance sensing in an optical microcavity. *Nature* **548,** 192–195 (2017).
5. Loh, K. P., Bao, Q., Eda, G. & Chhowalla, M. Graphene oxide as a chemically tunable platform for optical applications. *Nat. Chem.* **2,** 1015–1024 (2010).
6. Roy, R., Hohng, S. & Ha, T. A practical guide to single-molecule FRET. *Nat. Methods* **5,** 507–516 (2008).
7. Chen, J.-L., Yan, X.-P., Meng, K. & Wang, S.-F. Graphene oxide based photoinduced charge transfer label-free near-infrared fluorescent biosensor for dopamine. *Anal. Chem.* **83,** 8787–8793 (2011).

8. Liu, S.-H., Tang, W.-T. & Yang, Y.-H. Adsorption of nicotine in aqueous solution by a defective graphene oxide. *Sci. Total Environ.* **643,** 507–515 (2018).
9. He, S. *et al.* A graphene nanoprobe for rapid, sensitive, and multicolor fluorescent DNA analysis. *Adv. Funct. Mater.* **20,** 453–459 (2010).
10. Yao, B. C. *et al.* Partially reduced graphene oxide based FRET on fiber-optic interferometer for biochemical detection. *Sci. Rep.* **6,** 23706 (2016).
11. Zhang, Y., Wang, Y., Jia, J. & Wang, J. Nonenzymatic glucose sensor based on graphene oxide and electrospun NiO nanofibers. *Sensors Actuators B Chem.* **171–172,** 580–587 (2012).
12. Yi, J., Lee, J. M. & Park, W. Il. Vertically aligned ZnO nanorods and graphene hybrid architectures for high-sensitive flexible gas sensors. *Sensors Actuators B Chem.* **155,** 264–269 (2011).
13. Cao, S., Zhang, L., Chai, Y. & Yuan, R. An integrated sensing system for detection of cholesterol based on TiO2–graphene–Pt–Pd hybridnanocomposites. *Biosens. Bioelectron.* **42,** 532–538 (2013).
14. Mauranyapin, N. P., Madsen, L. S., Taylor, M. A., Waleed, M. & Bowen, W. P. Evanescent single-molecule biosensing with quantum-limited precision. *Nat. Photonics* **11,** 477–481 (2017).

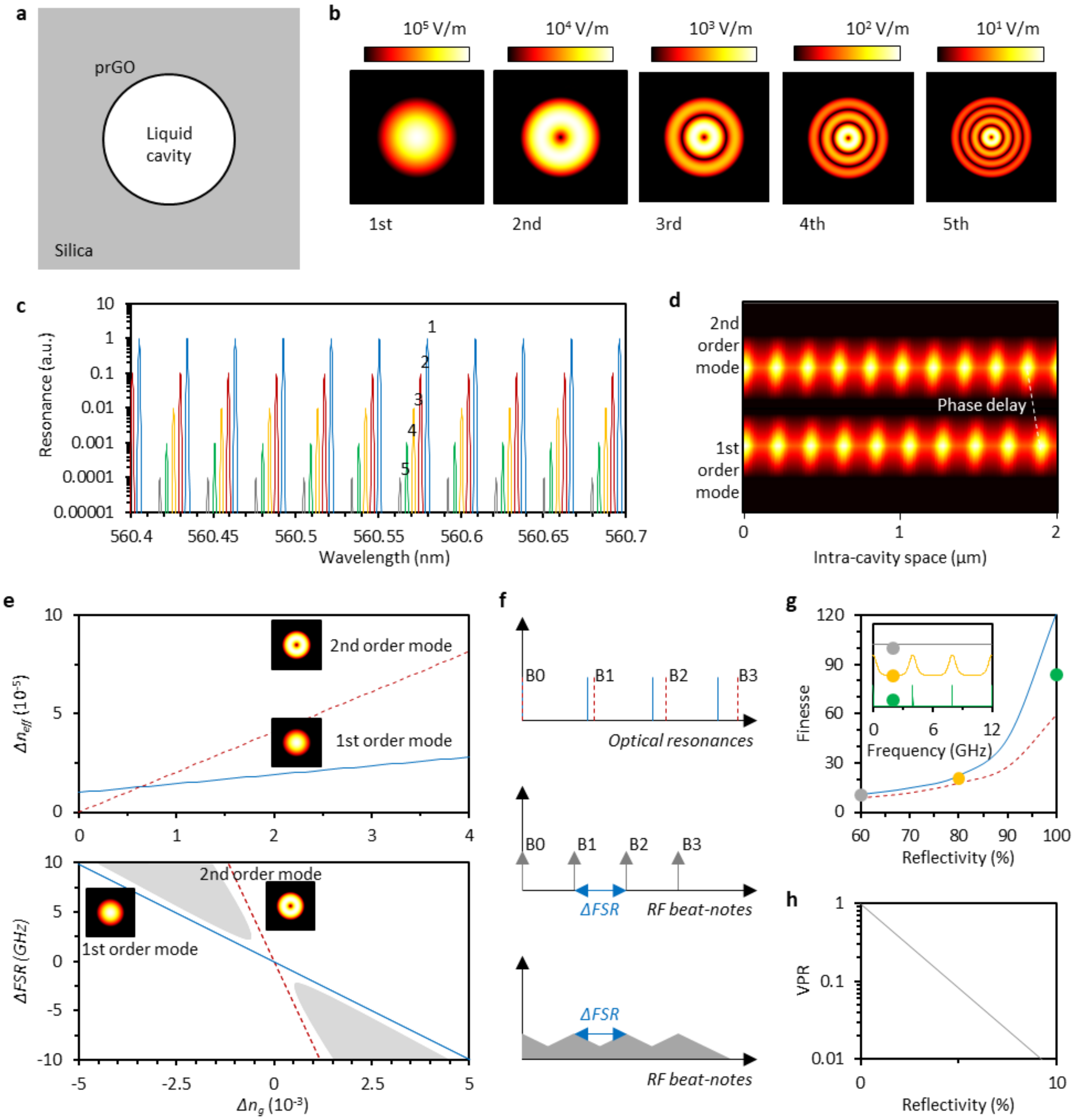

Fig. S1. Intracavity multiple transverse modes and their beating interference. a, Cross-sectional model of the resonator. **b,** Transverse modes, from order 1 to order 5. **c,** Longitude mode families of the transverse modes. Determined by the linear cavity resonance, intensity of higher order mode is smaller. Here the mode orders are marked. **d,** Spatial longitude mode distribution of the 1st mode and the 2nd mode, they have distinguished n_{eff}, enabling interference spectrally. **e,** Spectral sensitivity of the 1st order mode and the 2nd order mode, the difference brings spectral crossing and dividing. **f,** Mismatched FSR enables harmonic beatings in RF. Considering the inherent linewidth of the beating, wider ΔFSR or higher finesse is in need to avoid the spectral overlapping. **g,** By optimizing the quality of the reflectors thus increasing finesse, for fixed ΔFSR,

beating harmonics are more detectable. **h,** valley-to-peak rate of the beatings, the lower it is means the better detectability.

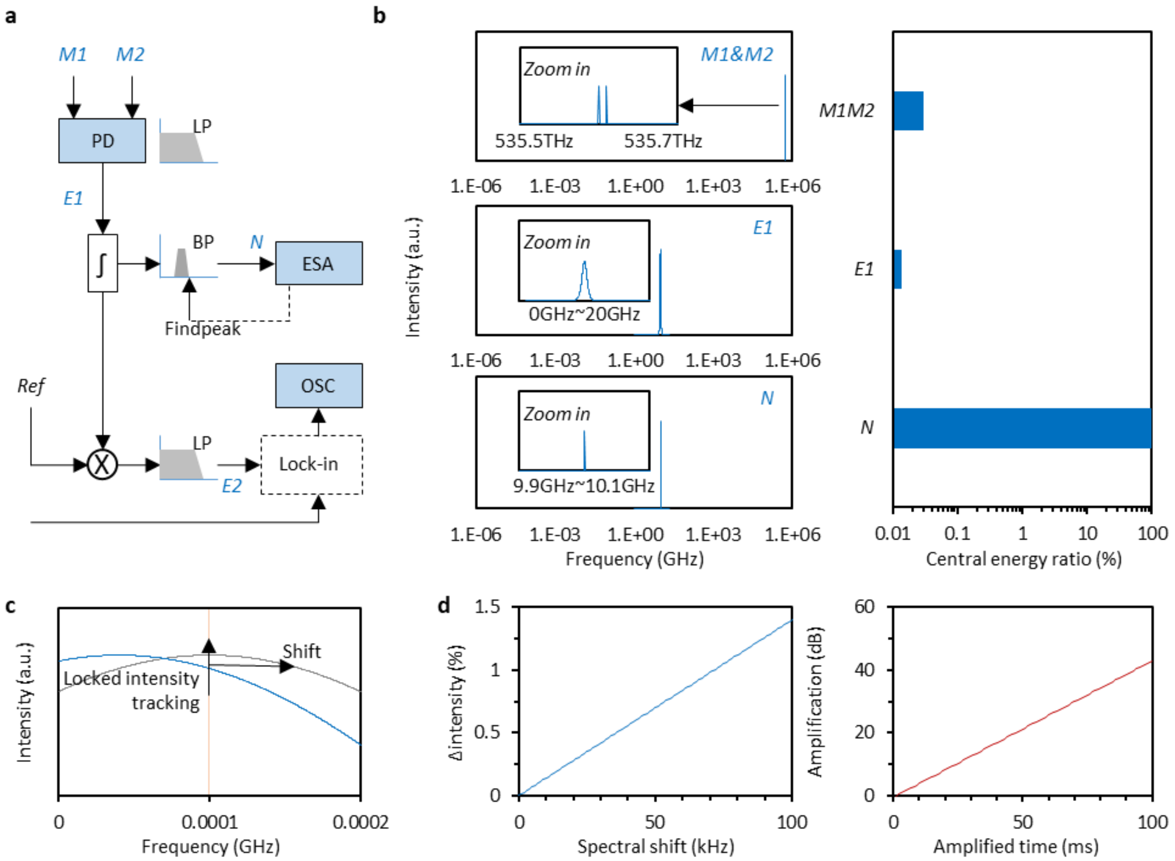

Fig. S2. Processing in electronics. a, Scheme of the high resolution beating and lock-in amplification. **b,** Spectral moving, from optics to electronics. By finding the peak and regenerating the ultranarrow beat note, spectral resolution can be remarkably enhanced. **c,** Spectral shift also brings intensity alteration at the fixed frequency in reference (e.g. at 100 kHz). **d,** For 700 MHz linewidth beat note, its intensity at the locked frequency changes 1.4% per 100 kHz shift. Such an alteration can be further amplified over 40 dB in the filter region.

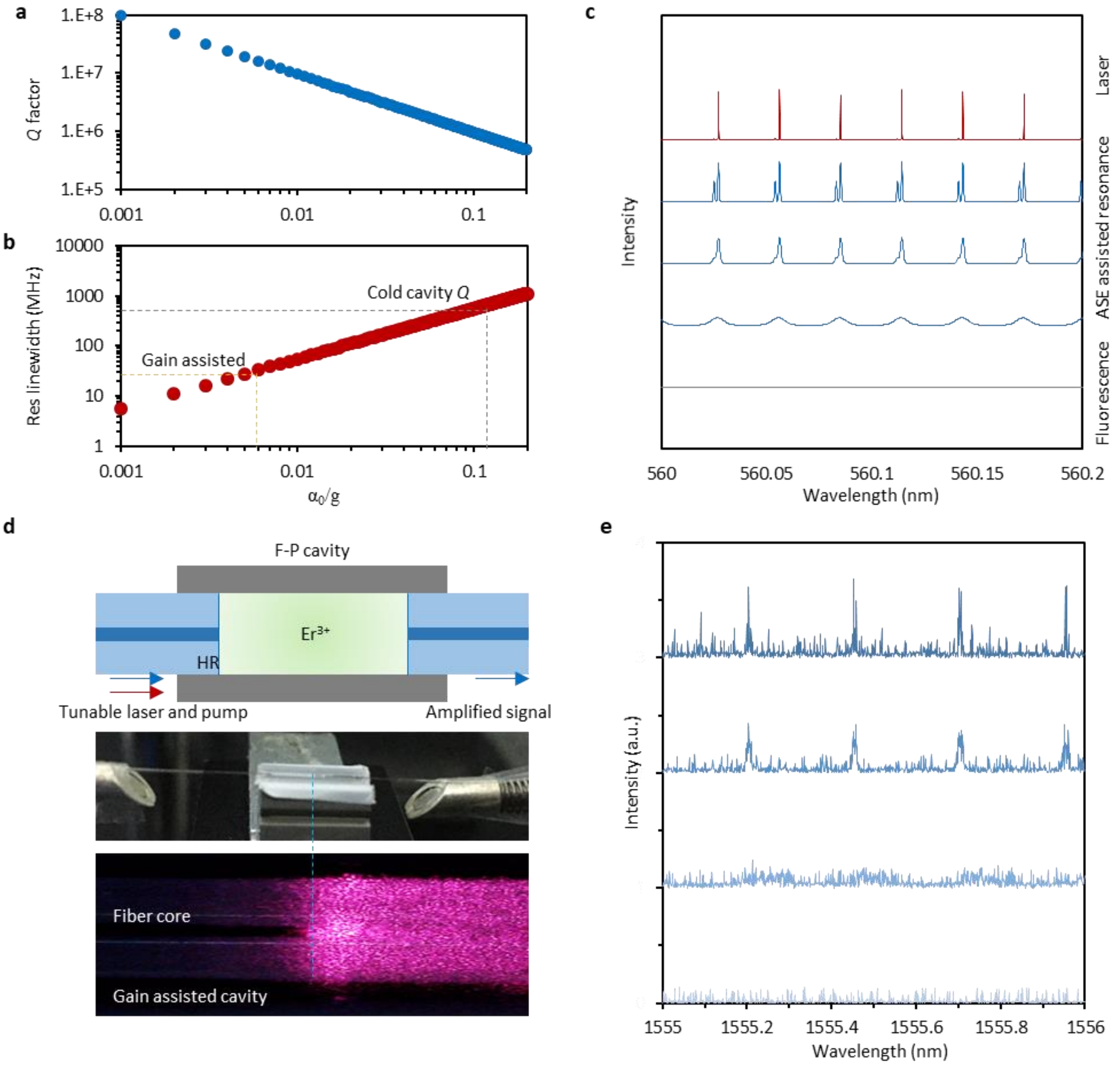

Fig. S3. Gain assisted resolution enhancement. a, By increasing the optical gain, the *Q* factor boosts. **b,** Correspondingly, by increasing the optical gain, the resonance linewidth can be suppressed by orders. **c,** By increasing the gain via boosting the pump power, resonances can be enhanced, calculated in visible band. **d,** Measuring the optical interference in a Er^{3+} based cavity, by using the high resolution (500 kHz linewidth) tunable laser in C band. **e,** Results show that the optical gain compensates loss of the open cavity, enabling the resonances via increasing the effective *Q*.

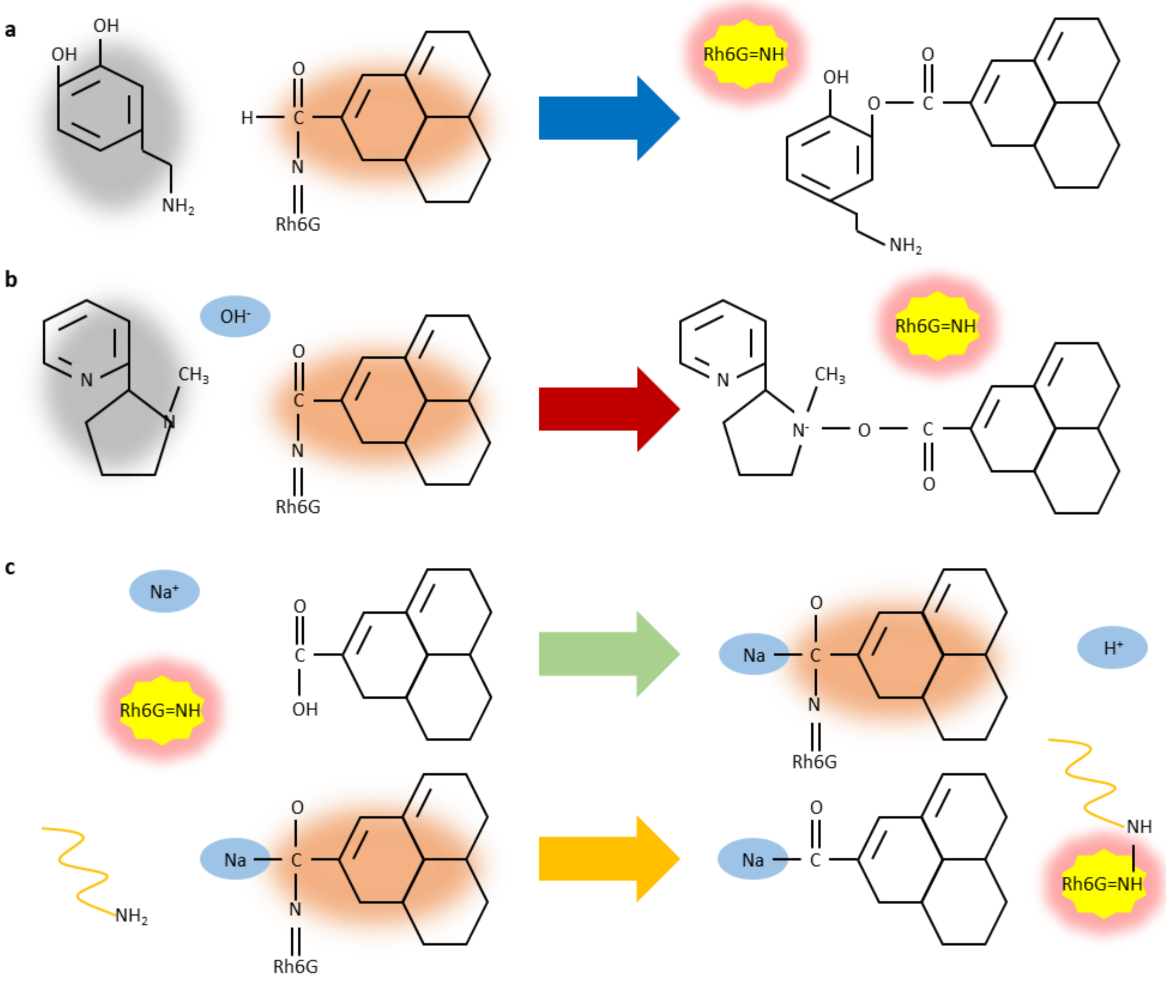

Fig. S4. Binding competitions. a, DA and prGO/Rh6G in acidic presetting. **b,** Nicotine and prGO/Rh6G in alkaline presetting, **c,** Functionalization of the Na^+/prGO$^-$/Rh6G, and the further FRET releasing Rh6G to the ssDNA.

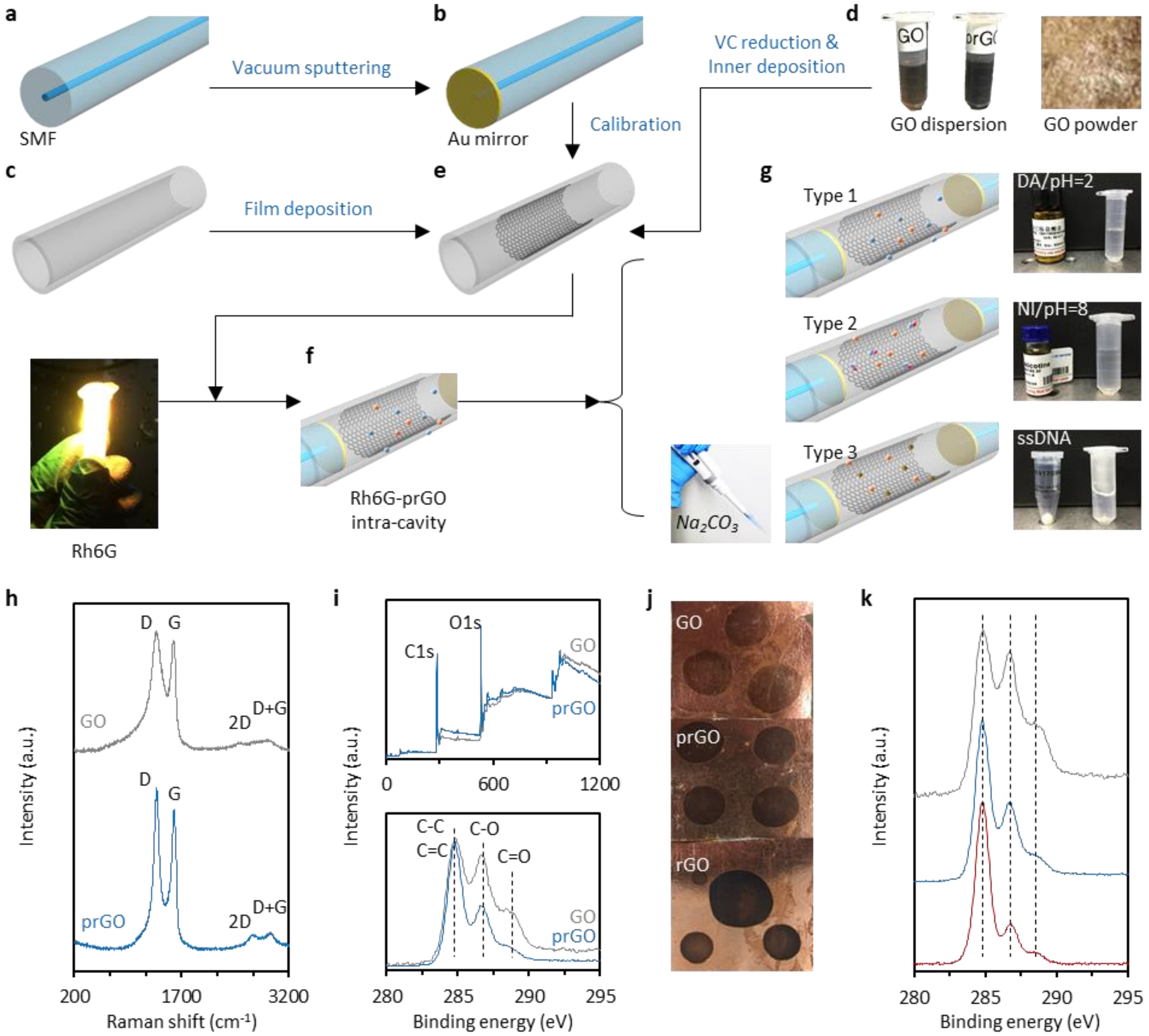

Fig. S5. Device fabrication and material characterization. a, Optical fiber. **b,** Au coating on the fiber end. **c,** Silica capillary. **d,** GO dispersion preparation and partial reduction. **e,** prGO deposition in the capillary. **f,** Rh6G deposited in the prGO based cavity. **g,** $H^+/OH^-/Na^+$ functionalization enables 3 types of sensors for DA, nicotine and ssDNA detection. **h,** Raman spectra, **i,** XPS spectra in band 0 to 1200 eV and 280 to 295 eV, showing that the reduction works. **j,** Pictures of the GO, prGO, and rGO deposited on copper foil. Their color represents their reduction degree. **k,** XPS of GO, prGO, and rGO.

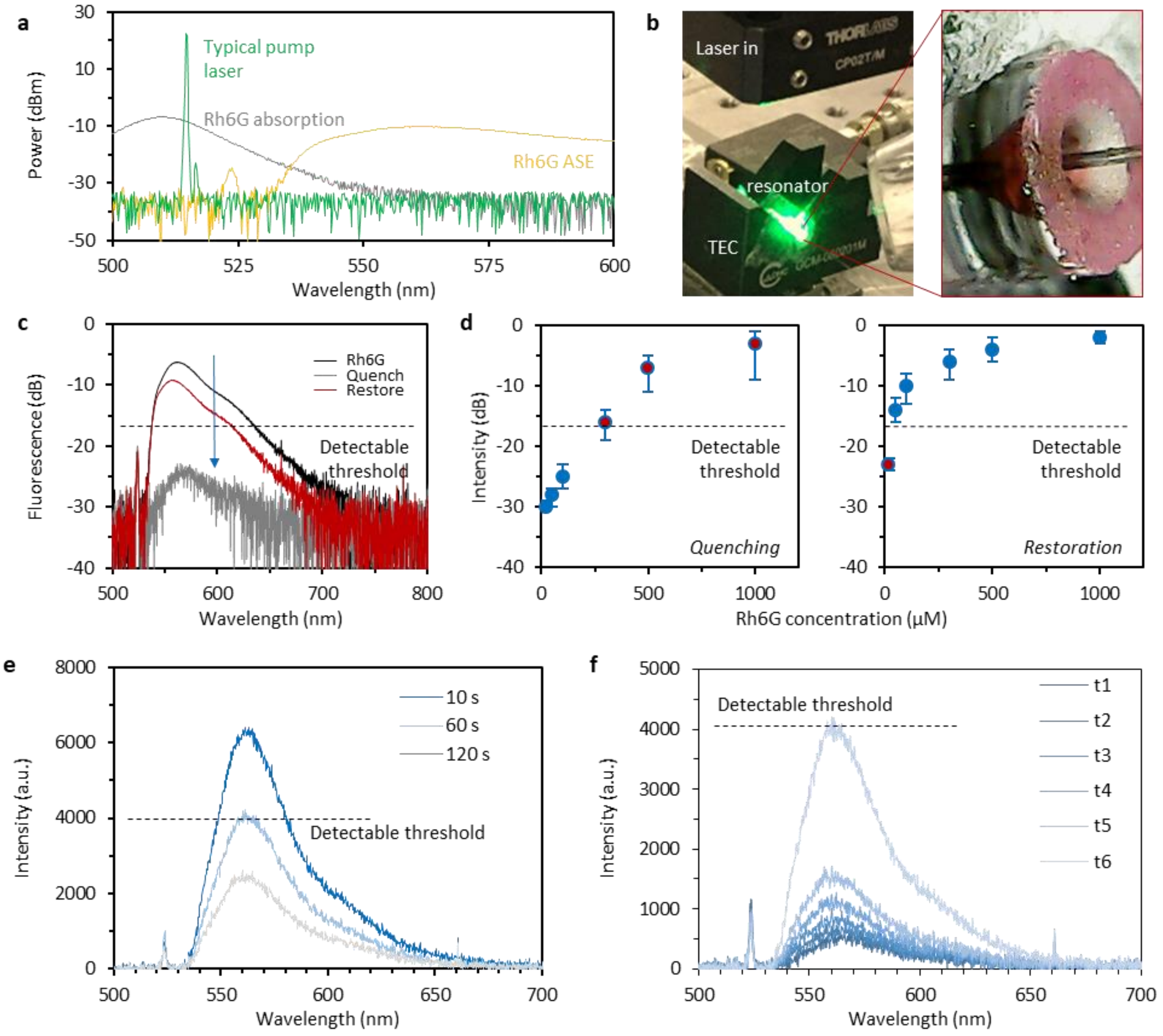

Fig. S6. Additional fluorescence measurement. a, the absorption and gain band of the Rh6G. We use a 514 nm CW pump laser for the fluorescent resonance generation. **b,** Picture of the F-P cavity in a holder, which is illuminated by the green laser pump, and well temperature controlled. **c,** Typical spectra of the fluorescence, the prGO in the capillary can fully quench 100 μM Rh6G, with quenched intensity 20 dB lower. **d,** The concentration of the Rh6G in pre-implementation influences the experimental effect. To achieve both good quenching and restoration for selectivity, we use 100 μM Rh6G in the fabrication. **e,** Bleaching wherein the fluorescent intensity decays gradually. **f,** With repeated use the quenching efficiency decreases. The sensor loses its selectivity after 6 times reloading.

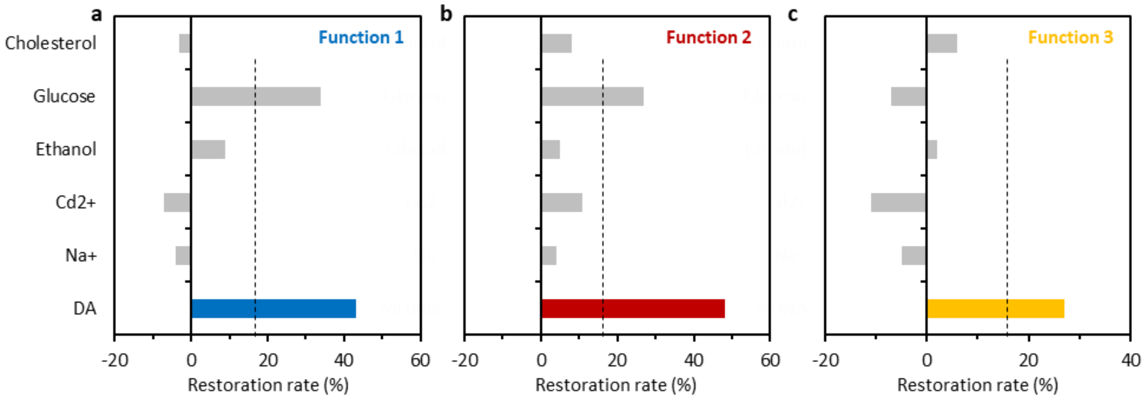

Fig. S7. Comparison of fluorescent restoration of extended analytes. The investigated biochemical molecules are Na^+, Cd^{2+}, ethanol, glucose, and cholesterol. **a,b, and c** shows fluorescent counts under the same Rh6G concentration 100 μM, in Function 1, 2 and 3 respectively. Here the dashed line indicates the detectable threshold for optoelectronic heterodyne beating.

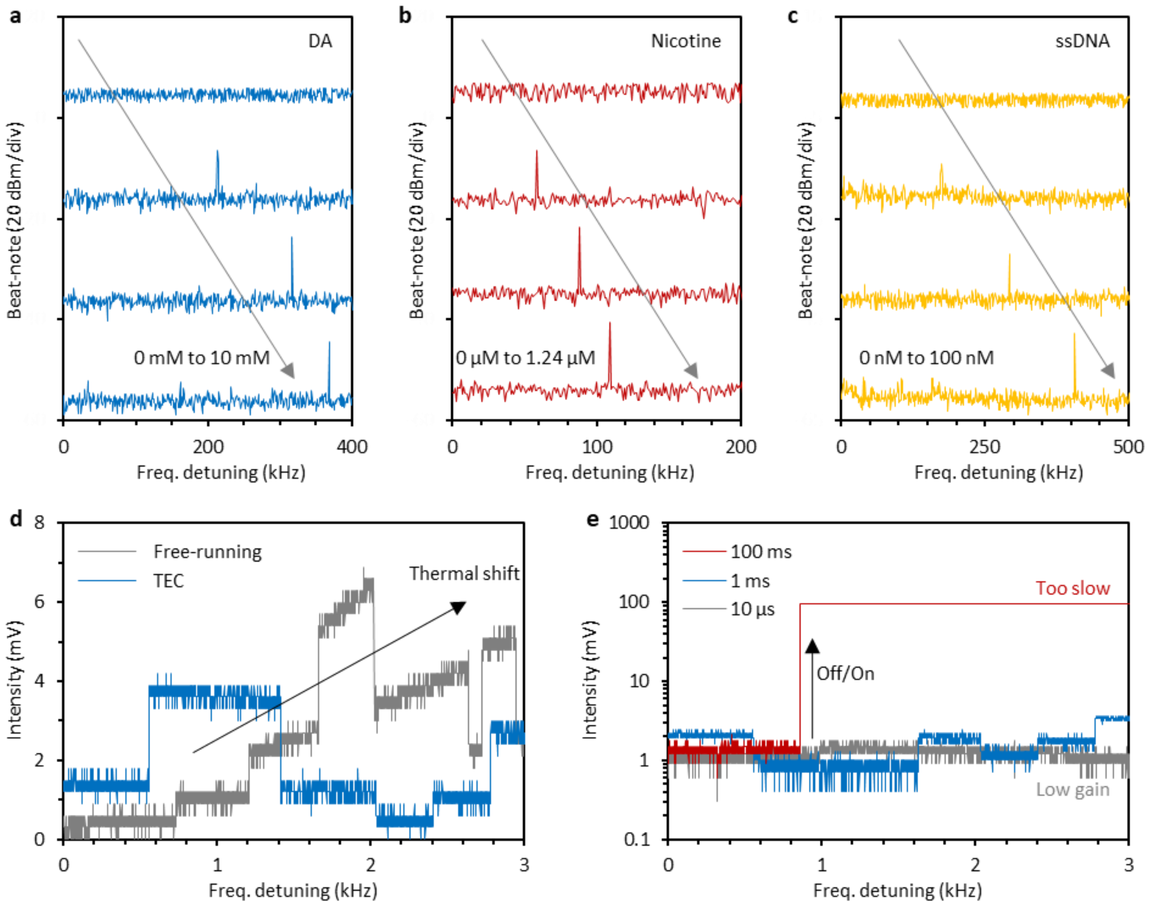

Fig. S8. Beat notes and lock-in traces in sensing. a, DA detection, by using sensors with function 1. **b,** Nicotine detection, by using sensors with function 2. **c,** ssDNA detection, by using sensors with function 3. The results meet the map shown in Fig. 3. **d,** Comparison of the free-

running sensor and the thermally stabilized sensor. Although the inter-mode interference is insensitive to the temperature in principle, the thermal shift in the material and the electronics would be amplified by the lock-in amplification. **e,** Comparison of different amplification rate. Proper amplification rate with proper speed is essential for tracking the molecular dynamics.

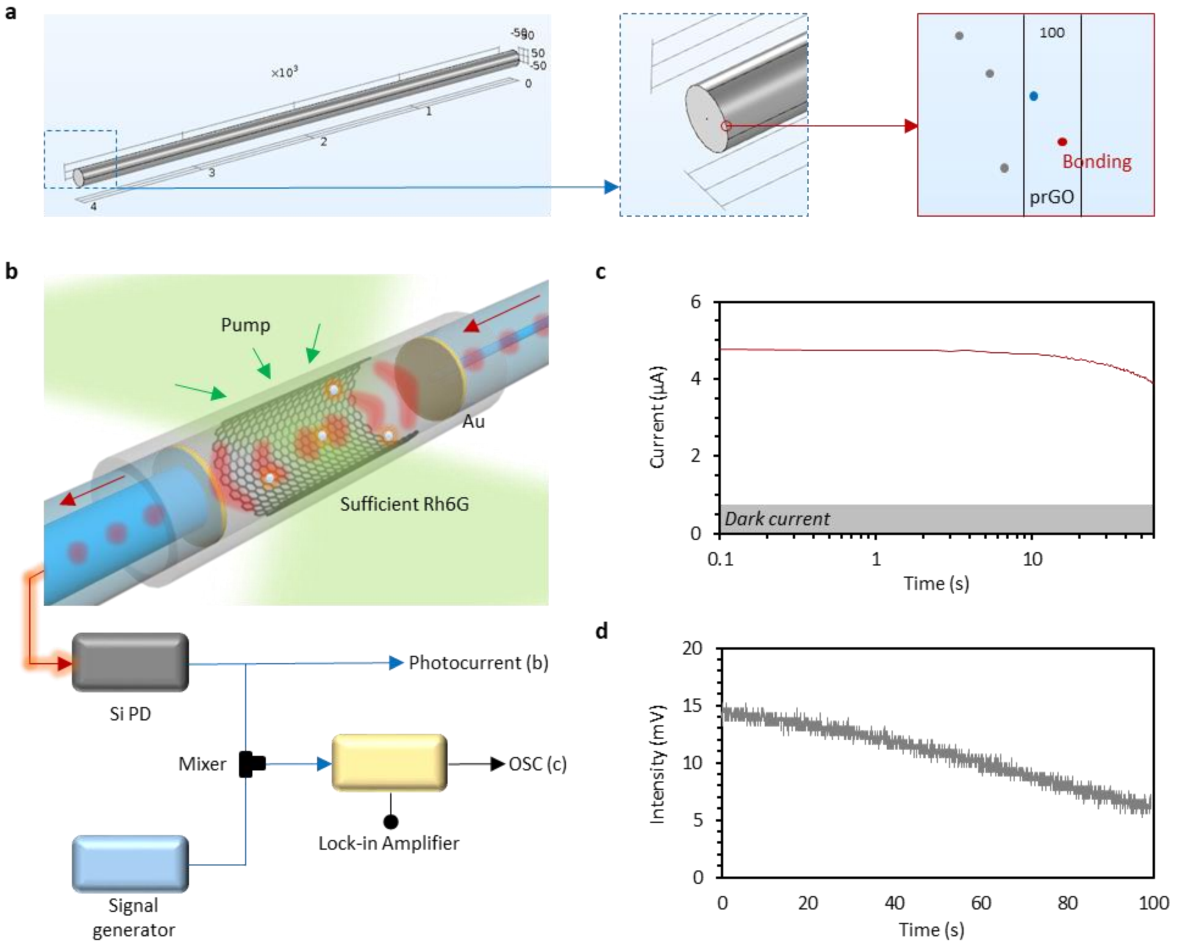

Fig. S9. Simulated Molecular distributions in the resonator and measurement of the resonator only filled with Rh6G. a, Simulation: Modelling and zoom-in. We implement the particle dynamics in commercial software *COMSOL Multiphysics*. **b,** Scheme and set-up measuring the Rh6G response only. **c,** Measured photocurrent in 1 minute duration. **d,** Temporal trace after the down-mixing and lock-in amplification.

Table S1. Estimation of the molecular activities at room temperature

	Number of molecules in cavity	Estimated number of molecules defusing onto the prGO	Estimated number of molecules bonded on the prGO	Estimated number of target-Rh6G bonding competition *per* min
**DA**	6×10^{13}	1.8×10^{11}	1.1×10^{4}	130
**Nicotine**	9×10^{9}	2.7×10^{7}	1.2×10^{4}	70
**ssDNA**	5×10^{8}	1.5×10^{6}	6×10^{3}	30

Table S2. Performance of the measurement

	Direct optoelectronic measurement	Filtering and lock-in amplification
**Theoretical photonic intensity sensitivity**	1.14×10^{4} photons per nA	50 photons per mV
**Detect resolution**	10 nA	0.1 mV
**Integration time**	0.1 s	0.001 s
**Rh6G quantum fluctuation (per photon)**	10^{-4} nA, Non-detectable	0.02 mV, Non-detectable
**DA induced increment *via spectral shift* (per molecule)**	Non-detectable	0.3 mV
**Nicotine induced increment *via spectral shift* (per molecule)**	Non-detectable	0.5 mV
**ssDNA induced increment *via spectral shift* (per molecule)**	Non-detectable	2.4 mV
